# Supplementary material for: Prognostic Role of Sonographic Decongestion in Patients with Acute Heart Failure with Reduced and Preserved Ejection Fraction: A Multicentre Study
Source: J Clin Med. 2023 Jan 18;12(3):773. doi: 10.3390/jcm12030773 (PMC9917462; doi:10.3390/jcm12030773)
Supplement: Supplementary file 1 [file jcm-12-00773-s001.zip › jcm-2156220-supplementary.pdf]

Supplemental Table S1. Univariate predictors of the primary end-point (cardiovascular death and re-hospitalization for heart failure).

Parameter	Univariate analysis HR (95% CI)	p-value
**Demographics**		
Age, years	0.99 (0.95 – 1.03)	0.5
Female gender	1.06 (0.95 – 1.24)	0.2
BSA (m^2)	0.31 (0.15 -1.62)	0.6
BMI (kg/m^2)	0.94 (0.79 – 1.11)	0.4
Family history of CVD	0.89 (0.20 – 4.01)	0.8
Diabetes mellitus	2.65 (0.87 – 8.07)	0.09
Arterial hypertension	0.37 (0.12 – 1.13)	0.08
Dyslipidaemia	2.61 (0.88 – 7.72)	0.08
CAD	2.04 (0.69 – 6.05)	0.2
Prior MI	2.17 (0.73 – 6.43)	0.1
Prior coronary revascularization	0.72 (0.09 – 5.59)	0.7
Atrial fibrillation	0.58 (0.39 – 1.18)	0.07
**In-hospital evaluation**		
Creatinine (mg/dL) at admission	1.51 (0.89 – 2.55)	0.1
eGFR ($mL/min/1.73m^2$) at admission	0.99 (0.98 –1.01)	0.3
NYHA class III-IV at admission	1.37 (0.50 – 3.77)	0.5
NT-proBNP (pg/mL) at admission	1.00 (1.00 – 1.01)	**0.01**
NT-proBNP (pg/mL) at discharge*	1.00 (0.99 – 1.01)	0.5
In-hospital diuresis (L)	1.00 (0.99 – 1.01)	0.8
Intravenous furosemide (mg)	0.99 (0.99 – 1.01)	0.9
Intravenous inotropes	1.09 (0.27 – 4.33)	0.9
Admission CXR		
Vascular congestion	1.47 (0.29 – 1.12)	0.7
Interstitial edema	2.3 (0.30 – 1.78)	0.4
Alveolar edema	3.27 (0.89 – 1.19)	0.07
Unilateral pleural effusion	2.72 (0.87 – 5.16)	0.08
Bilateral pleural effusion	0.80 (0.48 – 1.49)	0.8
**Home medications**		
Beta-blockers	2.63 (0.54 – 12.71)	0.2
ACE inhibitor/ARB	0.73 (0.15 – 3.49)	0.7
MRA	1.01 (0.25 – 3.99)	0.9
Furosemide	3.02 (0.35 – 11.71)	0.3
Furosemide dose (mg/die)	1.01 (0.99 – 1.01)	0.3
Thiazide / thiazide-like diuretics	2.29 (0.27 – 9.55)	0.4
Digoxin	0.66 (0.14 – 3.19)	0.6
Calcium-channel blockers	0.71 (0.09 – 5.65)	0.7
Amiodarone	0.95 (0.77 – 1.39)	0.3
Statins	0.50 (0.06 – 3.99)	0.5
Oral anticoagulants	0.80 (0.21 – 2.98)	0.7
Antiplatelet drugs	0.78 (0.09 – 6.19)	0.8
**Echocardiography at admission**		
EDV (mL/m^2)	0.97 (0.93 – 1.02)	0.2
ESV (mL/m^2)	1.04 (0.99 – 1.10)	0.07
LV ejection fraction (%)	1.03 (0.99 – 1.08)	0.09

LVMi (g/m^2)	0.99 (0.97 – 1.02)	0.8
Relative wall thickness	3.92 (0.87 – 9.04)	0.2
LAVi (mL/m^2)	0.97 (0.88 – 1.07)	0.6
Anteroposterior LAD (mm)	0.97 (0.91 – 1.04)	0.4
Mitral regurgitation	5.19 (1.80 – 14.9)	**0.002**
Mitral stenosis	2.37 (0.65 – 8.61)	0.2
Aortic regurgitation	2.67 (0.73 – 9.74)	0.1
Aortic stenosis	1.45 (0.25 – 11.3)	0.4
E-wave (cm/s)	1.01 (0.99 – 1.03)	0.1
A-wave (cm/s)	1.05 (0.97 – 1.15)	0.2
E/A ratio	0.73 (0.4 – 3.59)	0.7
Restrictive pattern[§]	0.57 (0.40 – 3.24)	0.6
RA minor axis (cm/m^2)	1.08 (0.99 – 1.16)	0.06
RVOT PLAX diameter (mm)	1.03 (0.94 – 1.13)	0.5
TAPSE (mm)	0.91 (0.79 – 1.03)	0.1
PASP (mmHg)	1.05 (0.91 – 1.23)	0.7
IVC expiratory diameter (mm)	1.15 (1.07 – 1.24)	**<0.0001**
Dilated IVC without collapse	1.25 (1.12 – 1.69)	**0.01**
**Lung ultrasound**		
B-lines at admission	1.01 (1.01 – 1.02)	**0.03**
B-lines at discharge	1.04 (1.02 – 1.06)	**<0.0001**
ΔB-lines%	1.01 (1.01 – 1.01)	**0.02**
Decongestion rate (ΔB-lines/die)	1.05 (0.95 – 1.15)	0.3

*available in 148/208 patients (71%).
See legend in Table 1 and 2.

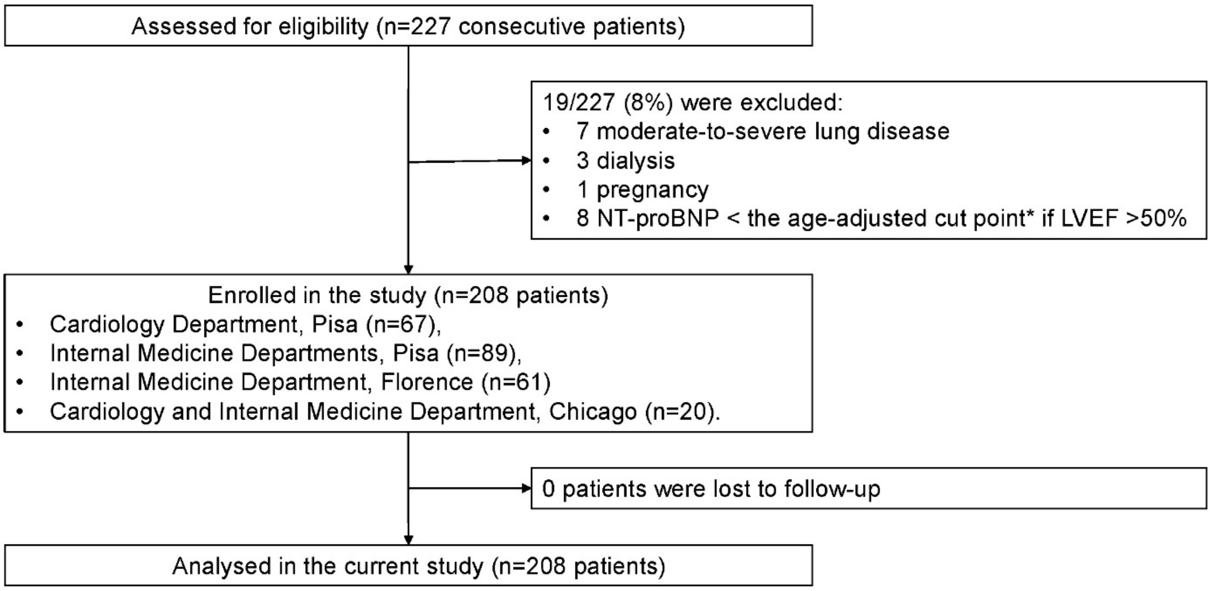

Supplemental Figure S1. CONSORT flow diagram. LVEF: left ventricle ejection fraction; NT-proBNP: N-terminal prohormone of brain natriuretic peptide.

* NT-proBNP ≤900 pg/mL ages 50-75, ≤1,800 pg/mL over age 75(14).

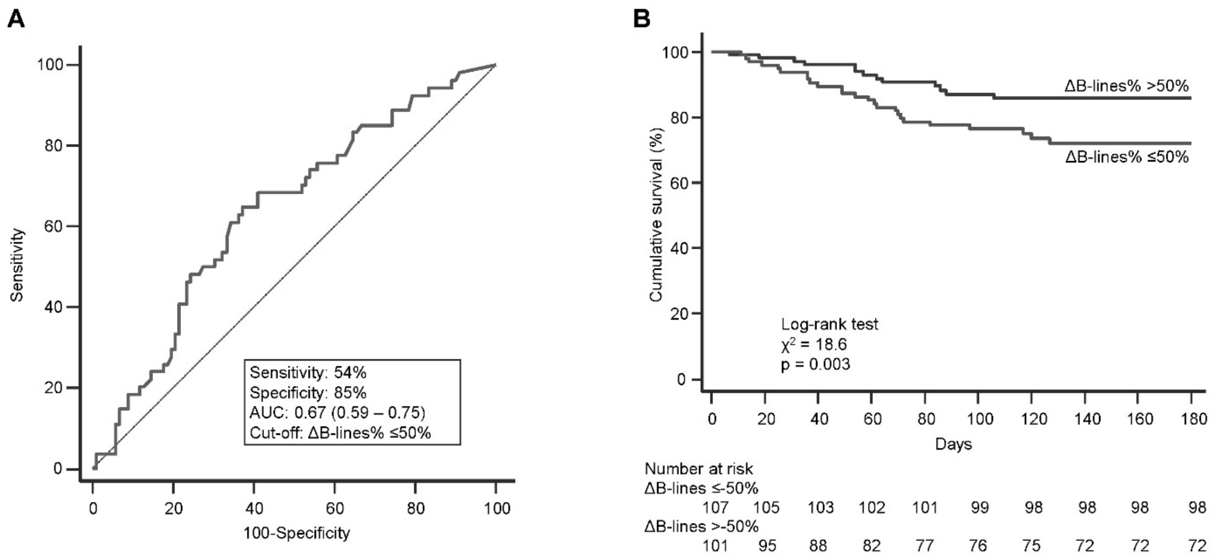

Supplemental Figure S2. **A) ROC analysis of ΔB-lines% in predicting the composite endpoint (cardiovascular death and rehospitalization for heart failure).** ROC curve analysis describing the performance of ΔB-lines% to identify the primary endpoint (rehospitalization for heart failure and cardiovascular death). The AUC and 95% confidence interval are shown, as well as the sensitivity and specificity at the cut-off identified based on the highest Youden index. ΔB-lines% = (B-lines at discharge – B-lines at discharge) / B-lines at admission %. AUC: area under the curve; ROC: receiver operating characteristic.

B) Kaplan-Meier survival curves stratified according to the ROC-derived cut-off. The curves illustrate a significant difference in cumulative survival, with patients having ΔB-lines% ≤50% experiencing a worse outcome than those with ΔB-lines% >50%. Numbers of patients at risk are shown below the survival curves.
